# Prevalence of depression and associated clinical and socio-demographic factors in people living with lymphatic filariasis in Plateau State, Nigeria

**DOI:** 10.1371/journal.pntd.0005567

**Published:** 2017-06-01

**Authors:** James Obindo, Jibril Abdulmalik, Emeka Nwefoh, Michael Agbir, Charles Nwoga, Aishatu Armiya’u, Francis Davou, Kurkat Maigida, Emmanuel Otache, Ajuma Ebiloma, Samuel Dakwak, John Umaru, Elisha Samuel, Christopher Ogoshi, Julian Eaton

**Affiliations:** 1 Department of Psychiatry, Jos University Teaching Hospital, Jos, Plateau State, Nigeria; 2 Department of Psychiatry, College of Medicine, University of Ibadan, Ibadan, Oyo State, Nigeria; 3 CBM Country Co-ordination Office, Federal Capital Territory, Abuja, Nigeria; 4 QHC, Department of Psychology, University of Jos, Jos, Plateau State, Nigeria; 5 The Carter Centre, Jos, Plateau State, Nigeria; 6 Health and Development Support Programme (HANDS), Jos, Plateau State, Nigeria; 7 CBM International and London School of Hygiene and Tropical Medicine, London, United Kingdom; Dodowa Health Research Centre, GHANA

## Abstract

**Background:**

Lymphatic filariasis is a chronic, disabling and often disfiguring condition that principally impacts the world’s poorest people. In addition to the well-recognised physical disability associated with lymphedema and hydrocele, affected people often experience rejection, stigma and discrimination. The resulting emotional consequences are known to impact on the quality of life and the functioning of the affected individuals. However, the management of this condition has focused on prevention and treatment through mass drug administration, with scant attention paid to the emotional impact of the condition on affected individuals. This study aimed to determine the prevalence and severity of depression among individuals with physical disfigurement from lymphatic filariasis in Plateau State, Nigeria.

**Methodology:**

A cross-sectional 2-stage convenience study was conducted at 5 designated treatment centers across Plateau State, Nigeria. All available and consenting clients with clearly visible physical disfigurement were recruited. A semi-structured socio-demographic questionnaire, Rosenberg Self-esteem and a 9-item Patient Health Questionnaire (PHQ-9) were administered at the first stage. Those who screened positive (with a PHQ-9 score of five and above) were further interviewed using the Depression module of the Composite International Diagnostic Interview (CIDI).

**Results:**

Ninety-eight individuals met the criteria and provided consent. Twenty percent of the respondents met criteria for depression, with the following proportions based on severity: Mild (42.1%), Moderate (31.6%) and Severe (26.3%). History of mental illness (OR 40.83, p = 0.008); Median duration of the illness was 17 years (IQR 7.0–30 years) and being unemployed (OR 12.71, p = 0.003) were predictive of depression. High self-esteem was negatively correlated (OR 0.09, p<0.004).

**Conclusion:**

Prevalence of depression is high among individuals with lymphatic filariasis and depression in sufferers is associated with low self-esteem and low levels of life satisfaction.

## Author summary

Lymphatic filariasis is a chronic illness that is disabling and often results in disfigurement. Affected people experience rejection, and stigma and discrimination, which can result in significant emotional consequences. Overall functioning and the quality of life of such individuals can be further affected by this exclusion and psychosocial impacts. Little or no attention is presently paid to the emotional impact of this disease in the overall management of people affected. The study, therefore, aimed to determine the prevalence and severity of depression, as well as associated socio-demographic factors, in individuals with physical disfigurement from lymphatic filariasis in Plateau State, Nigeria. Ninety-four consecutive consenting individuals with physically disfiguring lymphatic filariasis at 5 established treatment centers across Plateau State, Nigeria, were recruited and had semi-structured sociodemographic, Patient Health (PHQ-9), the **depression** module of Composite International Diagnostic Interview (CIDI) and Rosenberg Self-esteem questionnaires administered using a 2-stage design. Twenty percent of the sample were found to be depressed, while history of mental illness, duration of the illness, being unemployed, and religion were predictive of depression. High self-esteem was negatively correlated. The study underscores the need to go beyond just the physical needs of individuals with lymphatic filariasis. Management must be holistic and attention must be focused on the emotional sequelae of lymphatic filariasis.

## Introduction

Neglected Tropical Diseases (NTDs) are a group of disabling conditions that are among the most common infections affecting the world’s poorest people [1]. Many NTDs lead to chronic and often disfiguring conditions that result in significant disability and affect more than 1 billion people across the world [2].

Lymphatic filariasis (LF) is a mosquito-borne disease caused by filarial parasitic worms like *Wuchereria bancrofti*, *Brugia malayi and Brugia timori* [3]. Global estimates suggest that 120 million are affected in 80 countries throughout the tropics and subtropics with people at risk exceeding 1.3 billion [4, 5]. LF often manifests as enlargement of the entire leg or arm, the genitals (scrotal hydrocele in men), vulva and breasts leading to significant physical disfigurement [3]. Significant social stigma is associated with this stage of the disease [6], and the psychosocial problems linked with the condition are believed to be more severe than the physical ones [7].

In the 2010 Global Burden of Disease study, the Disability Adjusted Life Years (DALYs) associated with depressive illness in lymphatic filariasis was found to be twice that of the physical consequences of the disease (5.09 million global DALYs vs 2.78 million respectively) [8]. Despite this report, very few studies have been carried out to explore the association between depressive illness and lymphatic filariasis.

This study, therefore, aimed to determine the prevalence and severity of depression and the social and clinical factors associated with depression in individuals with lymphatic filariasis in Plateau State, North Central Nigeria.

## Methods

The study was carried out in five catchment areas in Plateau State where people living with LF periodically meet to receive physical care and support. These centers are run by the Carter Center, a US-based international Non-Governmental Organization (NGO). They include Tudunwada (Jos North Local Government Area (LGA)), Nyes (Mangu LGA), Ampier (Kanke LGA), Dadur (Langtang LGA) and Gwamlar (Kanam LGA). These sites include both rural and urban communities and were chosen as they have a high prevalence of LF.

The study population included all individuals accessing care at these five centers in Plateau State with obvious physical manifestation of the condition and who were available on site on the day of the visit of the research team. Advance notice was sent to all the Centers about a month before the visit, inviting potential participants to attend. There is no reason to believe that the attendees on these clinic days vary systematically from those attending on other days, though they are characterised by their access to clinical and rehabilitative care, which is not universal in Nigeria.

A cross-sectional 2-stage descriptive convenience study design was adopted. It involved the recruitment of all available and consenting individuals with physical manifestations of LF. Data was collected using a semi-structured socio-demographic questionnaire designed by the research team and a 9-item Patient Health Questionnaire (PHQ-9) for the first stage. Those who screened positive (with a score of 5 and above) were further interviewed using the Depression module of the Composite International Diagnostic Interview (CIDI) to make definitive diagnosis and rate the severity of the depression. Rosenberg Self-esteem scale was used to assess self-esteem in the study population.

On each day of the visit to a particular center, all participants were briefed on the purpose of the study. Each participant was then approached, given a more detailed explanation, consent obtained and the interview conducted in private. Six researchers (two consultant psychiatrists, a clinical psychologist, a social worker and two resident doctors in psychiatry) administered the socio-demographic, Rosenberg self-esteem and the PHQ-9 questionnaires to the participants and scored the PHQ-9. Those who scored 5 and above were sent to 2 consultant psychiatrists trained in the use of CIDI for the second stage. The participants were then formally diagnosed and rated for severity of the depression.

All those who screened positive for depression using the CIDI were given advice and information (psycho-education), and those who had moderate and severe depression were referred to a nearby comprehensive health facility for further treatment. They have subsequently been followed up in an on-going psychosocial service being developed at the Centers.

### Ethics statement

Ethical clearance for the study was obtained from the Institutional Research Ethical Committee of Jos University Teaching Hospital, Plateau State. Signed or thumb-printed written consent was individually obtained from each participant after due explanation of the purpose of the study and the voluntary nature of their participation. Respondents below the age of 18 years, required the consent of their parent or guardian in addition to their assent. The medical data as well as respondent scores on the instruments were anonymized in order to protect confidentiality.

### Data analysis

Data analysis was carried out using the Statistical Package for Social Sciences (SPSS), version 21, software using descriptive statistics to yield frequencies, percentages and proportions. Level of significance was kept at 5%. Logistic regression was used to identify predictors of depression among the respondents in this study using a few of the co-variates.

## Results

A total of ninety-eight participants, who met the inclusion criteria and gave consent for the study, were interviewed. Ninety-four (95.9%) had full documentation and were analysed. The majority, 58 respondents (61.7%), were female. Other socio-demographic details are provided in Table 1.

**Table 1:** Socio-demographic characteristics of study participants.

Characteristics	Frequency	Percentage
**Age group (years)**		
<18	3	3.2
19–30	10	10.6
31–40	9	9.6
41–50	15	16.0
51–60	18	19.1
>60	39	41.5
**Total**	94	100.0
**Sex**		
Female	58	61.7
Male	36	38.3
**Total**	94	100.0
**Marital Status**		
Married	48	51.1
Never married	13	13.8
Separated/Divorced/Widowed	33	35.1
**Total**	94	100.0
**Religion**		
Christianity	72	76.6
Islam	16	17.0
Traditional Worshipper	6	6.4
**Total**	94	100.0
**Employment Status**		
Full time	57	60.6
Part time	7	7.4
Retired	9	9.6
Unemployed+	21	22.3
**Total**	94	100.0
**Highest level of education**		
No formal	44	46.8
Primary	21	22.3
Secondary	17	18.1
Tertiary	10	10.6
Others*	2	2.1
Total	94	100.0
**Occupation**		
Artisan	3	3.2
Civil Servant	5	5.3
Farming	35	37.2
Student	4	4.3
Trading/Business	14	14.9
Unemployed	26	27.7
Others**	7	7.4
**Total**	94	100.0

* = Adult education

** = Driver, bike rider, security personnel etc., + = unemployed or current students

The median duration of illness was 17 years (IQR 7.0–30.0 years). Twenty-three (24.5%) had the illness for more than 30 years. 21 respondents (22.3%) rated their level of functioning as poor. Other details of functioning, as well as perceived adequacy of support are presented in Table 2.

**Table 2:** History of mental illness, economic status, functioning and adequacy of support.

Characteristics	Frequency	Percentage
**Family history of mental illness**		
Present	10	10.6
Absent	84	89.4
**Total**	94	100.0
**Previous history of mental illness**		
Present	2	2.1
Absent	92	97.9
Total	94	100.0
**Sources of income**		
Pension	2	2.1
Personal earning	62	66.0
Support from family/friends	30	31.9
**Total**	94	100.0
**Perceived adequacy of support**		
Adequate	32	34.0
Not adequate	62	66.0
**Total**	94	100.0
**Perceived adequacy of income**		
Adequate	17	18.1
Not adequate	77	81.9
**Total**	94	100.0
**Assessment of functioning**		
Poor	21	22.3
Average	56	59.6
Good	17	18.1
**Total**	94	100.0
**Income per month (Naira)**		
≤5,000	63	67.0
5,001–10,000	8	8.5
>10,000	23	24.5
**Total**	94	100.0
*Median monthly income (Naira) = N27*, *500 ($90*.16)****		
**Duration of illness (years)**		
1–10	33	35.1
11–20	28	29.8
21–30	10	10.6
>30	23	24.5
**Total**	94	100.0
*Median duration of illness = 17 years (IQR 7*.*0–30*.*0 years)*		

**** = it is highly skewed

**Fig 1:**
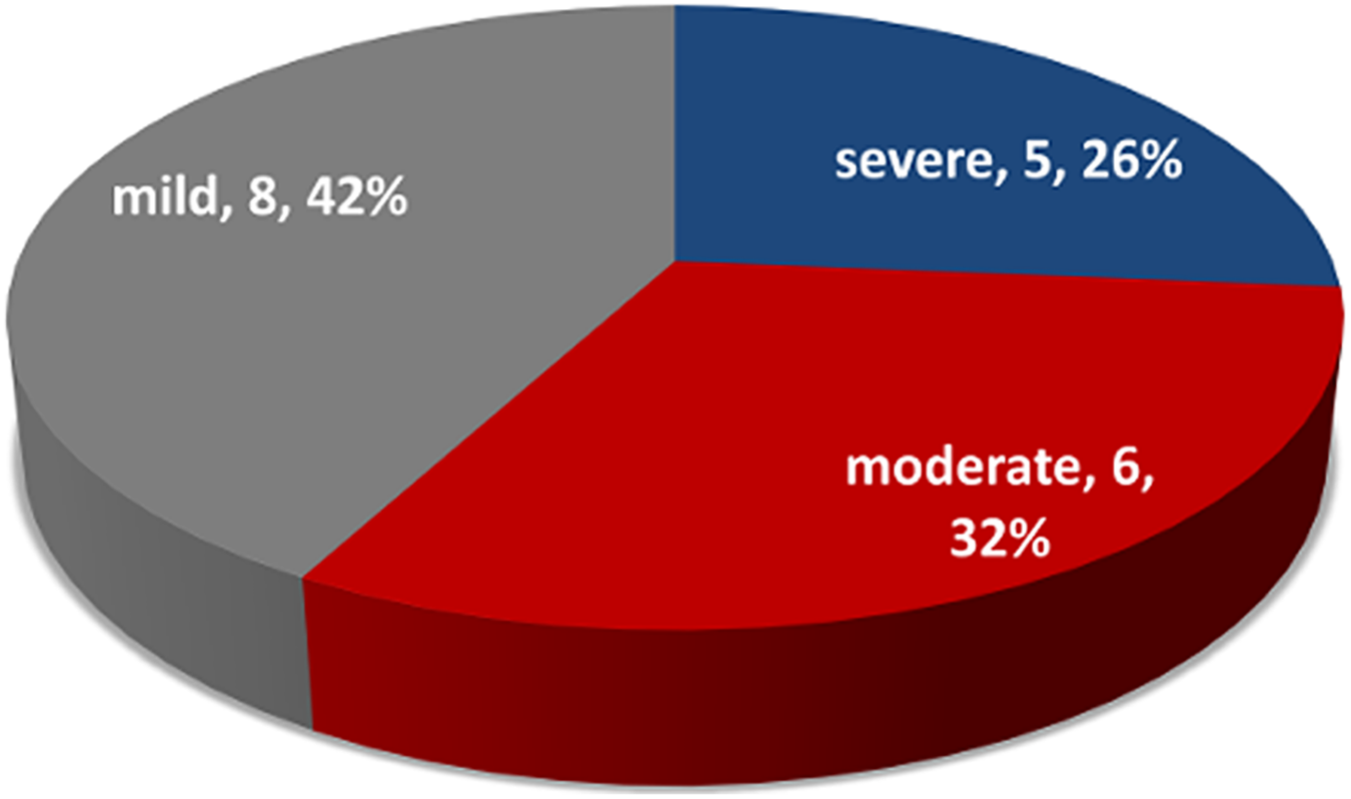
Severity of depression among respondents.

Nineteen respondents (20%) met criteria for depression, using CIDI, with the severity of the depression being Mild [8 (42.1%)], Moderate [6 (31.6%)] and Severe [5 (26.3%)]. See Fig 1.

Fourteen of the depressed respondents (73.7%) were female. Furthermore, 69 respondents (73.4%) reported low self-esteem. See Table 3.

**Table 3:** Prevalence of depression and low self-esteem.

Characteristics	Frequency	Percentage
**Diagnosis**		
Depressed	19	20.0
Not depressed	75	79.8
**Total**	94	100.0
**Self Esteem Status**		
High self esteem	25	26.6
Low self esteem	69	73.4
**Total**	94	100.0

Logistic regression analysis revealed that history of mental illness (OR 40.83, p = 0.008); duration of the illness between 11–20 years (OR 5.02, p = 0.079), being unemployed (OR 12.71, p = 0.003) and Self Esteem (OR 0.09, p = 0.004) were predictive of depression in the cohort. High self-esteem was negatively correlated to depression. See Table 4 below.

**Table 4:** Predictors of depression.

Factors	odds ratio	95% Confidence interval	P-value
**Family history of Mental illness**			
Present	0.63	0.005–7.7860	0.715
Absent	1	-	-
**Previous history of Mental illness**			
Present	40.83	1.6300–5.3403	0.008*
Absent	1	-	-
**Self esteem**			
High	0.09	0.0183–0.4594	0.004*
Low	1	-	-
**Duration of illness (years)**			
11–20	5.02	0.8299–30.4181	0.079
21–30	1.41	0.0951–20.8282	0.804
> 30	4.71	0.6693–33.0767	0.120
1–10	1	-	-
**Employment status**			
Part time	1.25	0.1351–11.5473	0.845
Retired	0.00	0.000 >1.0E12	0.964
Unemployed	12.71	2.4510–6.7005	0.003*
Full time	1	-	-

*Statistically Significant, though sample size was too small to assess the impact

## Discussion

Lymphatic filariasis cuts across all age-groups affecting both the elderly and the young with a slight preponderance in the mid to older age group. The over-representation of women (61.7%) in the study population is in keeping with the population of people receiving care at the Centers. The percentage of the participants who were separated/ divorced was higher than the expected in the general population (35.1% as against 6.8% in the general population) [9] and is in keeping with typical findings among this study population in relevant literature. They are less likely to be married, often described as the “last choice” in qualitative studies, and when the disease manifest itself during their married life, it may lead to divorce (7, 8). Nearly half of the study population (46.8%) had no formal education but this is not a sharp departure from typical rates in the rural population of the country [9]. The participants were largely self-employed as farmers and traders, which reflects the usual occupation of the people in the study areas. The average monthly income of more than three-quarter of the study population was less than N10, 000 (<$50 monthly) and this was described by more than 80 percent of the participants as inadequate. This is in keeping with the fact that NTDs principally impact the world’s poorest populations, as well as being a driver to poverty by itself [1]. The median duration of illness was 17 years (IQR 7.0–30.0 years), with about a quarter being over 30 years, illustrating relatively early onset and the chronic nature of the condition.

The percentage of those found to be depressed among the people with lymphatic filariasis (20%) is higher than the reported prevalence of depression among adults in Nigeria (3.1–5.2%) [10, 11, 12] and in primary care patients [13]. It is, however, similar to reported prevalence of depression in chronic medical conditions [14], who have been found to have two- to three- fold higher rates of major depression when compared with age- and gender- matched primary care patients [15, 16].

More women were found to be depressed among the study population, with a female to male ratio of almost 3:1. This large difference cannot be explained by the higher representation of women (F:M ratio of 1.6:1) in the study sample, and is also higher than the reported two-fold greater prevalence of Major Depressive Disorder in women than men in the general population [17]. This may be associated with greater social exclusion or stigmatisation experienced by women than men.

History of mental illness is an important and recognised risk factor, as such individuals are at greater risk of a relapse or development of another mental illness. Individuals with previous history of mental illness are almost 41 times more likely to be depressed than those without a previous history in our study.

Participants with low self-esteem, as measured by the Rosenberg self-esteem questionnaire in this study, were more likely to have depression when compared to those with high self-esteem. This could be attributable to the fact that people with depression are more likely to have low self-esteem. Our study, and many others in the region [18] and elsewhere have found that low self-esteem is often seen in people with NTDs possibly as a consequence of self-stigma, discrimination and limitations in functioning. This would suggest that high self-esteem is protective against the development of depression among this group. This is in keeping with recognised mechanisms linking stigma and depression, and may point towards potential therapeutic or health promotion interventions [19]. The link between social stigma and mental illness, including depression, are reported to be cyclical and reinforcing [19]. Self-stigma, a self-imposed restriction of expectations is common and may negatively affect expectations and motivation to engage socially [20]. In fact, people affected by LF will experience both the stigma associated with their physical condition and of their comorbid mental health problems.

Duration of the illness may be associated with higher prevalence of depression for a number of reasons. This may include the chronicity of exposure to risk factors inherent in the condition (disfigurement, pain, disability) which would be further compounded by the severity of the illness (likely to be greater in longer-term illness). It is also possible that there will be a greater extent of social drift over time. In keeping with this, unemployment was also found to be associated with greater prevalence of depression. This is the pattern found with many psychiatric disorders, where there is a vicious cycle of mental illness provoking poverty (through mechanisms summarised as social drift) and poverty being a risk factor for mental illness (social causation, or poverty as a social determinant) [21].

### Limitations

1. The sample method in the study, being a convenience sampling technique, is a major limitation. The study population being from a treatment center potentially excludes those in the community who are not accessing care, and may not be representative of the total relevant population. A community study would address this problem, though it is likely that the most physically affected individuals are those most likely to be accessing services.
2. The sample size is relatively small. This limits generalization and may have affected the analysis of co-variates.

## Conclusion

Beyond the physical burden of living with lymphatic filariasis, people with the condition also have significant psychiatric complications (particularly depression). Appropriate treatment for depression has been found to improve outcomes in other chronic conditions [22]. Therefore, given the high prevalence of depression, providing access to mental health screening and interventions should be integral to NTD programmes. Thus, in addition to attending to physical needs, emotional needs should also be routinely assessed and catered for.

It is recommended that staff should be sensitised to the high risk of depression and be trained to recognize basic signs and symptoms of depression. Simple screening instruments for depression, such as the PHQ-9 can also be utilized in routine clinics, and those found to be depressed can be treated or referred appropriately. Health promotion strategies geared towards assessing and addressing factors associated with depression (for example self-esteem) should be incorporated in routine community engagement, including health talks in the clinic. These are simple steps that can easily be incorporated into specific NTD services, and general health care for endemic populations, providing the possibility of improving the quality of life of affected persons, and reducing the negative impact of depression on an already marginalised population.

## Supporting information

S1 STROBE checklist (DOC)
